# Echocardiography and cardiac resynchronisation therapy, friends or foes?

**DOI:** 10.1007/s12471-015-0769-3

**Published:** 2015-12-08

**Authors:** W.M. van Everdingen, J.C. Schipper, J. van ’t Sant, K. Ramdat Misier, M. Meine, M.J. Cramer

**Affiliations:** Department of Cardiology, University Medical Centre Utrecht, Heidelberglaan 100, PO Box 85500, 3508 GA Utrecht, The Netherlands

**Keywords:** Cardiac resynchronisation therapy, Echocardiography, Volume, Optimisation, Dyssynchrony, Response, Follow-up, Interobserver variability, Speckle tracking echocardiography, Deformation imaging, 3D echocardiography, Septal strain

## Abstract

Echocardiography is used in cardiac resynchronisation therapy (CRT) to assess cardiac function, and in particular left ventricular (LV) volumetric status, and prediction of response. Despite its widespread applicability, LV volumes determined by echocardiography have inherent measurement errors, interobserver and intraobserver variability, and discrepancies with the gold standard magnetic resonance imaging. Echocardiographic predictors of CRT response are based on mechanical dyssynchrony. However, parameters are mainly tested in single-centre studies or lack feasibility. Speckle tracking echocardiography can guide LV lead placement, improving volumetric response and clinical outcome by guiding lead positioning towards the latest contracting segment. Results on optimisation of CRT device settings using echocardiographic indices have so far been rather disappointing, as results suffer from noise. Defining response by echocardiography seems valid, although re-assessment after 6 months is advisable, as patients can show both continuous improvement as well as deterioration after the initial response. Three-dimensional echocardiography is interesting for future implications, as it can determine volume, dyssynchrony and viability in a single recording, although image quality needs to be adequate. Deformation patterns from the septum and the derived parameters are promising, although validation in a multicentre trial is required. We conclude that echocardiography has a pivotal role in CRT, although clinicians should know its shortcomings.

## Introduction

**Fig. 1:**
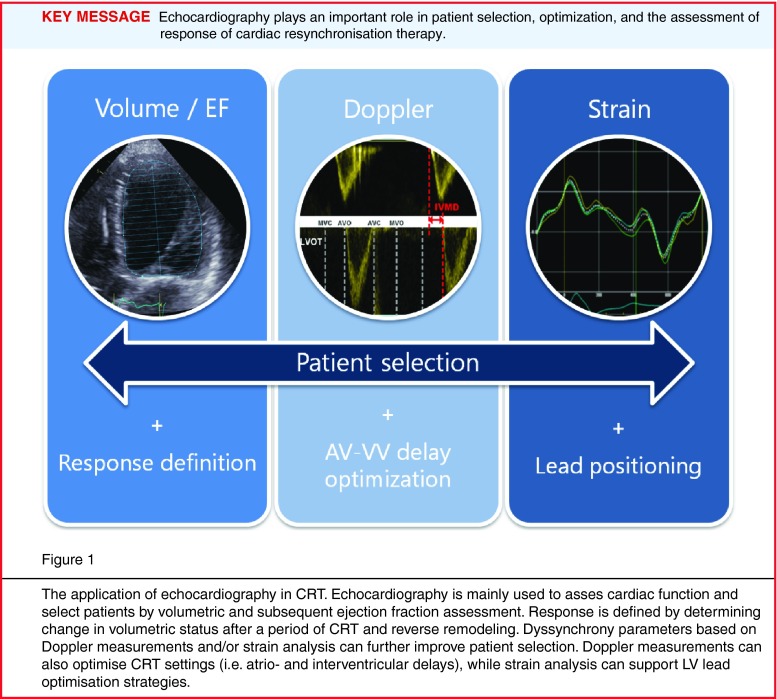
The application of echocardiography in CRT. Echocardiography is mainly used to asses cardiac function and select patients by volumetric and subsequent ejection fraction assessment. Response is defined by determining change in volumetric status after a period of CRT and reverse remodeling. Dyssynchrony parameters based on Doppler measurements and/or strain analysis can further improve patient selection. Doppler measurements can also optimise CRT settings (i.e. atrio- and interventricular delays), while strain analysis can support LV lead optimisation strategies

Thus far, echocardiography has a pivotal role in cardiac resynchronisation therapy (CRT), underlined by the wide field of application, determining cardiac function and specifically left ventricular (LV) function and response due to desired reverse electro-mechanical remodelling. The range of tools, from brightness mode or Doppler imaging to deformation imaging, offers the possibility of patient selection and response prediction, lead placement optimisation strategies and optimisation of device configurations (Fig. 1; [1–3]). Multiple single-centre studies have advocated the value of echocardiography in patient selection and determining prognosis [4–6]. However, limitations are known, as the PROSPECT study showed a limited value regarding response prediction, and EchoCRT gave insight into the potential negative effects of echocardiographic parameters as selection criteria [3, 7]. Moreover, echocardiography can have a relatively substantial measurement error and not every patient is suitable for adequate echocardiographic volume assessment, especially in non-expert hands [8]. The question arises whether echocardiography is a useful imaging tool for evaluation of CRT patients, or are its shortcomings impeding clinical decision making? This review reflects on the role of echocardiography in the field of CRT, are they friends or foes?

## Patient selection

### Left ventricular ejection fraction

Echocardiography gives insight into the cardiac anatomy and valvular dysfunction of CRT patients. Its main role in CRT is determining cardiac function and especially LV volumes and ejection fraction (LVEF). International guidelines on CRT define a cut-off for LVEF at ≤ 35 %, independent of the imaging tool used [9, 10]. A meta-analysis of randomised trials on the effects of CRT on morbidity and mortality has underlined this cut-off. A reduced benefit or even adverse effect in patients with an LVEF above the cut-off was observed, although the large confidence interval might indicate that a subgroup of patients with LVEF > 35 % do benefit (hazard ratio for all-cause mortality: 0.28–2.00) [11]. This could be due to an overestimation of LVEF by echocardiography. A sub-analysis of the PROSPECT study advocated the benefit of CRT in patients above the threshold [12]. The threshold for response to CRT is probably more a continuum than binary.

Although the biplane Simpson’s method is the most robust method to determine LV volume and function for echocardiography, intraobserver and interobserver variability can be high, with reported differences in LVEF of up to 18 % (Bland-Altman limits of agreement or two standard deviations) [8]. A study compared LVEF determined by a recruiting centre to an echocardiography core lab. The correlation coefficient was fair among 413 patients (R^2^: 0.69). A mean difference of 0.2 % was found, although a wide confidence interval was observed (95 % CI: − 17.4–17.8 %). Moreover, 20 % of all patients would have been reclassified by another centre, using a cut-off for LVEF of 30 % [13]. These results underline the limitations of echocardiography for a strict cut-off, beside the need for core lab activities.

Volumes derived with echo are underestimated compared with ‘gold standard’ magnetic resonance imaging (MRI) [14, 15]. Nevertheless a meta-analysis found a mean difference between the two modalities of close to 0 %, although a large spread and heterogeneity between studies was observed [8]. Assessment of LVEF by MRI and echocardiography shows opposing results for CRT eligibility in 28 % of patients, using the guideline cut-off. Compared with MRI, echocardiography underestimated both end-diastolic and end-systolic volume, while overestimating LVEF [15]. As most large multicentre trials on the selection of patients for CRT used echocardiographic-derived volumes, cut-offs cannot be directly translated to other imaging techniques.

Averaging of several measured beats improves accuracy in general, and is applicable to all patients and specifically in atrial fibrillation. Contrast-enhanced echocardiography for volume assessment can further reduce intraobserver and interobserver variability [16, 17]. An intravenous contrast agent can identify the endocardial borders more precisely. When used in either two- or three-dimensional echocardiography, LV volumes determined by contrast are also more similar (less underestimated) to MRI [16]. Results on LVEF are conflicting, as both similar and improved correlations to MRI are reported using contrast-enhanced volumes compared with conventional echocardiography [16–18].

Based on these findings, the cut-off for selection of patients eligible for CRT should depend on the imaging tool. Moreover, subgroups for LVEF 30–40 % could be incorporated in future guidelines [12]. Patients with LVEF above the current cut-off, but with a true left bundle branch block (LBBB), might benefit from CRT. Implementation of subgroups, in concordance with the role of QRS width in current guidelines, may reflect the role of LVEF in CRT more appropriately. However, evidence supporting CRT in subgroups based on LVEF needs to be further established.

### Dyssynchrony

Selection criteria for patients eligible for CRT based on electrical or mechanical dyssynchrony show a preference for an electrical substrate [9]. Correcting mechanical dyssynchrony without an electrical substrate has proven to be ineffective, as has been shown in large trials such as Echo-CRT [7]. Moreover, after PROSPECT, it remains debated whether mechanical dyssynchrony is warranted as an indicator for response to CRT, although the study design and applied dyssynchrony parameters are disputed [19]. Guidelines have so far been restricted to clinical and electrocardiographic selection criteria (i.e. QRS width and LBBB morphology). As previously mentioned, imaging tools are strictly necessary to determine LVEF. Mechanical dyssynchrony proven by any echocardiographic parameter is not included in current guidelines. Patients with a class I indication (i.e. LBBB) will most certainly have a form of mechanical dyssynchrony when assessed by echo. Patients with a class III indication (i.e. QRS < 120 ms) and with proven dyssynchrony are no better or even worse with CRT [7, 20]. The additional value of dyssynchrony parameters might be in the class IIa or b groups. In these groups, with prolonged QRS width (> 120 or > 150 ms) and non-LBBB, visualised mechanical dyssynchrony could indicate a treatable substrate.

Mechanical dyssynchrony parameters (Table 1) should be based on a physiological principle, where early septal and delayed activation of the LV free wall causes disturbed LV intraventricular and interventricular interaction. An ideal parameter indicates a substrate that can be corrected by biventricular pacing. Prediction models for volumetric response (> 15 % decrease in end-systolic volume) advocate the use of mechanical dyssynchrony parameters, on top of existing criteria [21, 22]. However, multicentre trials and meta-analysis have failed to show an added value of several dyssynchrony indices, although interventricular mechanical dyssynchrony (IVMD) was associated with increased survival in the CARE-HF study [3, 23, 24]. Other parameters (systolic rebound stretch of the septum (SRSsept), septal flash and apical rocking) are only proven in single-centre studies [4, 25, 26].

**Table 1:** Echocardiographic parameters for patient selection and/or response prediction

Parameter	Brief description	Echocardiographic image	Cut-off	Pros	Cons	Remarks
Apical rocking	Visual assessment of apical rocking motion	AP4CH view^a^	Yes/no [6, 25]	Easy method	Translating continuous process to an on/off phenomenon	Requires multicentre validation
					Confounded by RV-function	
Septal flash	Visual assessment of short septal motion during beginning of systole	AP4CH view, zoomed on septum or PLAX	Yes/no [26]	Easy method	Interobserver differences	Requires multicentre validation
					Translation of continuous process to on/off phenomenon	
IVMD	Interventricular mechanical delay, difference in onset of outflow of LV (LVPEP) and RV (RVPEP)	Pulsed-wave Doppler of LVOT and RVOT	40 ms [3, 11, 21]	Relatively easy method	Ambiguous results in multicentre trials	Still indicates probability of response
					Confounded by both LV and RV function	
						50 ms cut-off used in CARE-HF trial [28]
SRSsept	All positive deflections after initial shortening of the septum during systole	AP4CH view suitable for speckle tracking (B-mode) zoomed and focus on septum	4.7 % [4, 21, 31, 38]	Predicts volumetric response and outcome	Relatively difficult for non-trained personnel	One trial found 4.0 % as cut-off using entire AP4CHimages [32]
					GE specific	
					Interobserver differences	
						Requires multicentre validation
Septal strain patterns	Strain pattern of the septum during systole	AP4CH view suitable for speckle tracking (B-mode) zoomed and focus on septum	3 types (1 and 2 often responder, 3: often non-responder) [33, 34]	Predicts volumetric response and outcome	Relatively difficult for non-trained personnel	Requires multicentre validation
SL delay	Difference of time to peak velocity (or shortening) of (basal) septal and lateral wall	AP4CH view suitable for speckle tracking (B-mode) or TDI velocity delay	≥ 65 ms [3, 5]	Predicts volumetric response and outcome	Negative results in multicentre trial	
					Large influence of sampling Time-to-peak based	
SD-TTP	Standard deviation of time to peak shortening (strain) or velocity (TDI) off all myocardial segments	AP4CH, AP2CH and APLAX view suitable for speckle tracking (B-mode) or TDI	≥ 32 ms [3]		Time-to-peak based	Requires multicentre validation
					High image quality needed	
SDI	Time to minimal systolic volume of 16 segments	3D echocardiography	9.8 % [39]	High predictive value for response	Limited spatial and temporal resolution	Requires multicentre validation

*AP4CH* apical four chamber view*, B-mode* brightness mode, *GE* General Electric, *IVMD* interventricular mechanical delay, *LV* left ventricle, *LVOT* left ventricular outflow tract, *LVPEP* left ventricular pre-ejection period, *PLAX* parasternal long axis, *SDI* systolic dyssynchrony index, *SD-TTP* standard deviation of time to peak shortening, *RV* right ventricular, *RVOT* right ventricular outflow tract, *RVPEP* right ventricular pre-ejection period, *SL-delay* septal to lateral wall delay of time to peak shortening, *SRSsept* systolic rebound stretch of the septum, *TDI* tissue Doppler imaging.

**Fig. 2:**
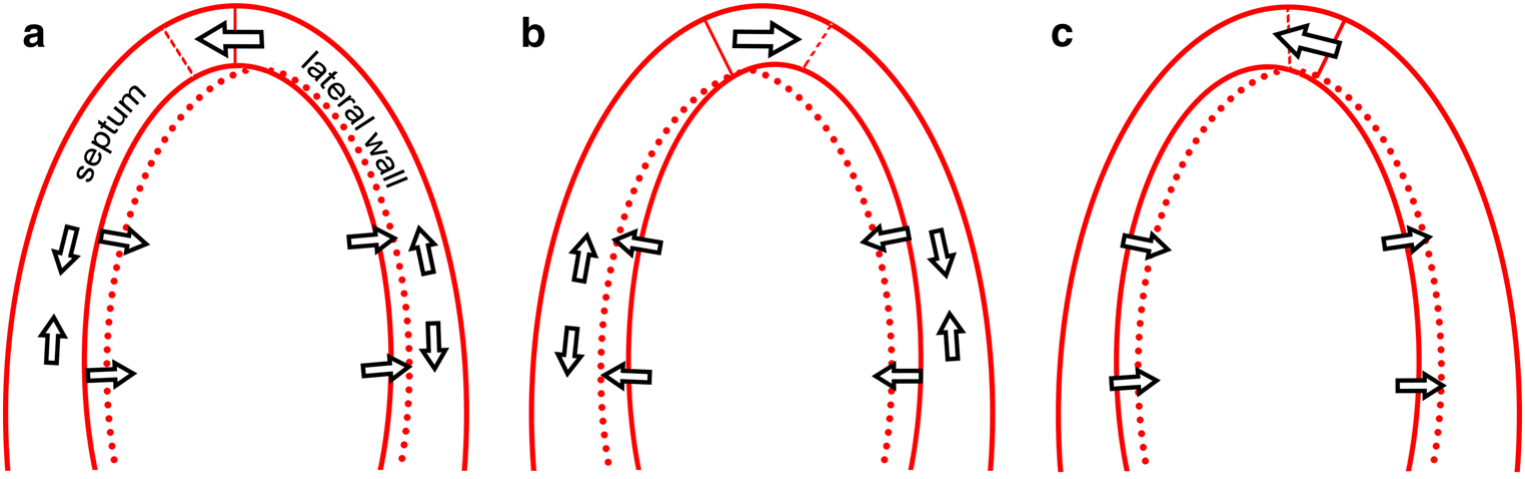
Central illustration. Role of echocardiography in CRT. Echocardiography can be used to select patients by volume and subsequent ejection fraction assessment and by dyssynchrony parameters based on Doppler and/or strain analysis. Doppler can also optimise CRT settings, while strain analysis could support LV lead optimisation strategies

Visual assessment or ‘eye-balling’ of mechanical dyssynchrony is perhaps the most feasible method for routine clinical applications. Two parameters are known: apical rocking and septal flash (Fig. 2) Both are known to have a high specificity for predicting response [25, 26]. Apical rocking is the apical transverse motion due to an inhomogeneity of myocardial contraction and function, and requires an apical four chamber view with a visible apex. LBBB causes early septal contraction which moves the apex towards the septum, while delayed lateral wall contraction subsequently ‘rocks’ the apex towards the lateral side. Szulik et al. proved the predictive value for volumetric response, with comparable strength for both visually assessed and automatically quantified apical rocking [25]. Ghani et al. confirmed these results and further showed that apical rocking predicts long-term clinical outcome in terms of hospitalisation for heart failure [6]. Septal flash shows similar results. A septal flash is a short inward septal motion, occurring due to early septal contraction, interrupted by delayed free wall contraction. Parsai et al. found the presence of septal flash to be predictive for both clinical and volumetric response [27]. On top of known predictors, the value of septal flash in prediction models was significant [22].

IVMD is the delay in onset of outflow between the left and right ventricle. Delayed activation and subsequent contraction of the LV free wall due to LBBB leads to a delayed rise in LV pressure and outflow. LV dyssynchrony therefore lengthens the LV pre-ejection period, increasing IVMD, while ‘normal’ right ventricular (RV) activation leads to a fast rise in pressure and outflow through the RV outflow tract. IVMD can, however, be confounded by reduced RV function and a lengthened RV pre-ejection period. It can be easily measured using pulsed-wave Doppler signals obtained in any standard echocardiogram. IVMD reflects dyssynchrony of interventricular dynamics, by subtracting the difference in onset between QRSonset and flow through both the left and right ventricular outflow tract (LV and RV pre-ejection period, respectively). Although a cut-off of 40 ms is used to determine dyssynchrony, IVMD has a linear relationship with response, and a specific cut-off for response is unsuitable [28]. It is therefore not applicable for patient selection, although the probability of response can be determined.

### Deformation imaging

Deformation imaging or strain analysis with echocardiography uses either tissue Doppler imaging or speckle tracking echocardiography. The latter is less angle dependent and covers the whole ventricular wall, in contrast to tissue Doppler imaging, and is therefore more reliable for detection of delayed activated segments.

**Fig. 3:**
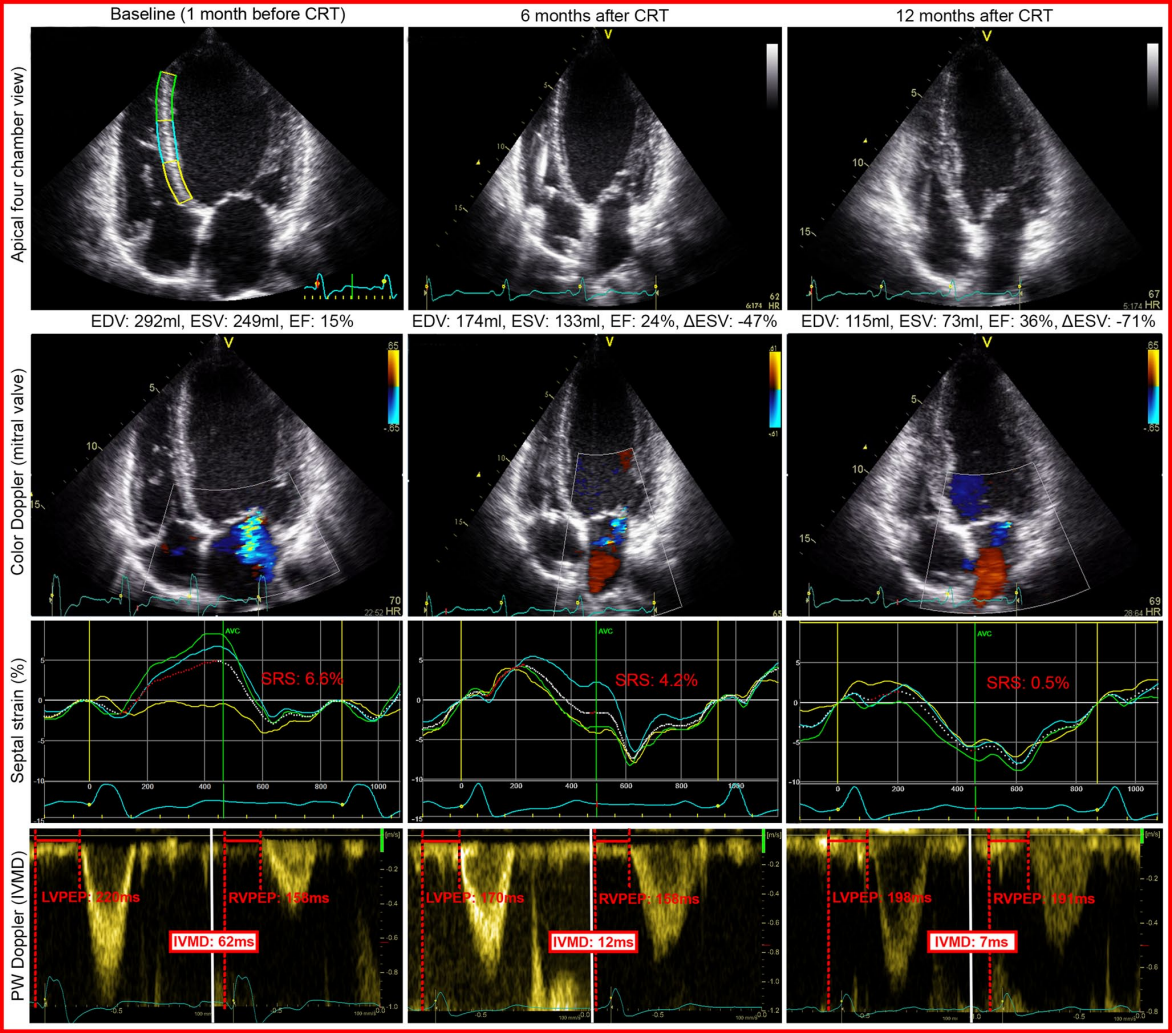
Schematic representation of apical rocking and septal flash. Schematic representation of the *left* ventricle in echocardiographic apical four-chamber view, showing both septal flash and apical rocking due to LBBB-induced mechanical dyssynchrony. **a** Early septal contraction stretches the lateral wall and rocks the apex to the *left*, while the septum thickens and moves inwards. **b** Late lateral wall contraction stretches the septum and rocks the apex to the *right*. **c** Relaxation of the lateral wall with continuing septal contraction, while the apex moves to its original position

Septal to lateral wall delay (SL delay) is obtained by either tissue Doppler velocity imaging or speckle tracking echocardiography, calculated by the time difference between peak velocity (Doppler) or contraction (Doppler and/or speckle tracking) of the basal septal and lateral wall [5]. SL delay thereby reflects both early septal and late lateral wall contraction caused by delayed free wall activation. Using tissue Doppler imaging, SL delay is measured by sampling in the basal septal and lateral wall, which is sensitive to sample placement [29]. As for all time-based parameters, defining the maximum peak is of importance. The maximal peak can be early or late, depending on the interaction with tethering myocardium and ventricular dynamics. Septal deformation can have a late maximum peak, while contraction starts early, resulting in an earlier first peak (baseline septal strain in Fig. 3). A dyssynchronous ventricle can therefore be deemed synchronous. Moreover, despite the promising results of single-centre studies, so far no multicentre trial has proved the diagnostic power of SL delay [3].

Most deformation parameters use time delays in peak contraction, either absolute, relative or as a standard deviation, to determine evidence of dyssynchrony [30]. These parameters suffer from the same definition-based limitations as SL delay. Parameters that incorporate the entire strain curve are therefore more promising. Moreover, high-quality images are required to determine segmental differences, which is not always feasible, especially in patients with dilated ventricles due to heart failure. The interventricular septum is therefore of particular interest, as echocardiographic imaging of the septum is almost always feasible and reproducible. Its central position in the ultrasound window guarantees adequate image quality. Moreover, the septum provides information on interventricular interaction as well as intraventricular properties. Derived parameters, such as SRSsept or identification of septal strain patterns, are promising as predictors of outcome (Fig. 3 and Table 2; [4, 31, 32]). SRSsept is the total amount of systolic rebound stretch, after initial shortening of the septum. Early septal shortening disrupted by contralateral delayed free wall contraction causes rebound stretch. SRSsept thereby assesses the amount of wasted work for the septum that can be recruited by CRT. Septal dyssynchrony can show multiple typical patterns, of which Leenders et al. discriminated three types [33]. These patterns (and SRSsept) are even influenced by myocardial viability and predict both clinical and volumetric response to CRT (Fig. 3; [33, 34]). Risum et al. recently demonstrated the role of LV strain pattern recognition on top of electrocardiographic predictors for outcome [35]. However, results depend on the ultrasound machine and speckle tracking echocardiography software used. The majority of algorithms to determine myocardial strain are unknown and lack validation. Inter-vendor differences are known and limit translation to other ultrasound machines than the most commonly published, i.e. GE EchoPac, General Electric Healthcare, Milwaukee, USA [36]. Intraobserver and interobserver coefficients of variation are relatively high for SRSsept (19.5 and 16.3 %, respectively) [4]. Despite the small number of clinical trials, all agree on the potential strength of septal strain parameters derived by speckle tracking echocardiography (Table 1; [32, 34, 37, 38]). A multicentre trial is required to prove their benefit in clinical decision making.

**Table 2:** Studies on septal dyssynchrony parameters predicting response to CRT

First author	Design	Subjects (*n*)	Parameter and cut-off	Response prediction (≥ 15 % ΔESV)	Outcome	Strengths and/or limitations
De Boeck [4]	Prospective single centre	62	SRSsept > 4.7 %	Sens/spec: 81 %/81 %, AUC: 0.938 ± 0.035, B: 2.41, *p* = 0.005		Relatively high inter- and intra-observer variability (COV: 14.2 and 15.6 %)
Leenders [31]	Prospective single centre	101	SRSsept > 4.7 %	Multivariate analysis, B: 3.78, *p* < 0.001	Survival (death, LVAD or transplant) with HR: 5.8 (2.3–14.3)	No HF hospitalisation or morbidity
Chan [38]	Prospective single centre	43	SRSsept > 4.7 %	AUC: 0.862 ± 0.061^a^		No multivariate analysis
van ’t Sant [21]	Retrospective single centre	227	SRSsept (continuous)	Multivariate analysis, B: 1.191		SRSsept assessed as continuous variable. No specific cut-off used
Ghani [32]	Retrospective single centre	138	SRSsept > 4.0 %	Sens/spec: 66 %/66 %, AUC: 0.70	Data on outcome not published (although registered)	Analysis on AP4CH instead of septal single wall
Leenders [33]	Retrospective single centre	132	Septal deformation patterns (3 types)	Type 1 and 2 predict response vs type 3 ΔESV: 37 ± 20 & 24 ± 24 vs 5 ± 20 ml, *p* < 0.001		Validated by mechanistic computer model
Marechaux [34]	Prospective single centre	101	Septal deformation patterns (3 types)	Responders: pattern 1&2 vs 3: 92 vs. 59 %, *p* < 0.0001, Sens/spec: 74 %/74 %	18 months event-free survival (death or HF hospitalisation): Pattern 1&2 vs. 3: 95 vs 75 %, *p* = 0.01	Relatively short follow-up
Risum [37]	Prospective single centre	67	LBBB deformation pattern	Sens/spec: 91 %/95 %		Complex pattern description
Risum [35]	Prospective multicentre	208	LBBB deformation pattern		Absence of LBBB-pattern increases 4 year risk of death, HF hospitalisation, LVAD or HTx HR 3.1 (1.64–5.88)	Complex pattern description

Studies on septal dyssynchrony parameters, derived from speckle tracking echocardiography, predicting response to cardiac resynchronization therapy. All studies are single centre, prospective trials.

*AUC* area under the curve in ROC analysis, *B* beta-coefficient, *COV* coefficient of variation, *ΔESV* difference in end-systolic volume*, HF* heart failure, *HR* hazard ratio, *HTx* heart transplantation, *LBBB* left bundle branch block, *LVAD* left ventricular assist device, *n* number of patients, *p* p-value, *sens* sensitivity, *spec* specificity, *SRSsept* systolic rebound stretch of the septum.

^a^when added to a model with clinical characteristics (gender, LBBB, QRS duration, heart failure aetiology).

Dyssynchrony can also be assessed by three-dimensional echocardiography (3DE). The predominant 3DE parameter is the systolic dyssynchrony index, using the standard deviation of difference from a reference time point in the QRS complex to minimal systolic volume of 16 segments. Systolic dyssynchrony index (mean cut-off 9.8 %) was able to predict treatment response with a sensitivity and specificity of 93 and 75 % respectively [39]. The intra-class correlation coefficients for intraobserver and interobserver variability were high (0.95 and 0.92 respectively). Nevertheless, the echocardiographic image quality (i.e. spatial and temporal resolution) needs to be adequate for analysis, which is not always feasible. QRS triggering should also be adequate, as triggering after QRS onset will miss early septal contraction, overestimate the onset and therefore underestimate time-to-peak values. Moreover, these parameters have been tested in single-centre studies, and therefore require validation in a multicentre trial. The diagnostic power of the systolic dyssynchrony index can therefore be overrated, as has been observed for previous parameters (e.g. SL delay) [3, 5].

## Optimising lead position

Radial strain obtained with speckle tracking echocardiography from parasternal short axis images prior to implantation can indicate segments with delayed peak contraction. During implantation, LV lead placement can be guided to these segments, resulting in a remote, adjacent (i.e. neighbouring), or concordant placement, based on the 17-segment model of the American Heart Association. Observational studies have shown that a concordant or adjacent position to the latest contracting segment is superior to a remote position, in terms of reverse remodelling, death and hospitalisation during 2 years of follow-up [40, 41]. Targeting the latest contracting segment with radial strain has been implemented in two randomised clinical trials, the TARGET and STARTER trial. Patients were randomised to targeted or conventional LV lead placement [1, 42]. Targeted placement led to a higher percentage of concordant or adjacent positions and showed improvement in both volumetric response (LV change in end-systolic volume, TARGET: − 30 ± 29 vs. − 20 ± 25 % and STARTER: − 46 ± 33 vs. − 26 ± 23 ml) and clinical outcome (percentage of patients reaching endpoint of death and hospitalisation for heart failure, TARGET: 22 vs. 42 % and STARTER: 14 vs. 21 %). A large number of leads in the unguided group were by chance placed in a favourable position. Per-protocol analysis of both studies showed that patients (guided or unguided) with concordant or adjacently placed leads had a better response and outcome [1, 42].

Another advantage of strain analysis is information on myocardial viability by peak strain values. Scarred regions are known to have lower strain amplitudes, and pacing in a region of scar tissue correlates to non-response [43]. Both abovementioned trials excluded segments with peak strain < 10 %, thereby excluding potentially scarred segments, which may have contributed significantly to the positive effects of echo-guided lead positioning. Sub-analysis showed that absence of scar near the LV lead was a strong predictor for volumetric response and reduced all-cause mortality in the TARGET trial [44]. Moreover, sub-analysis of the STARTER trial indicated that echo-guided LV lead placement improved survival especially in patients with ischaemic cardiomyopathy [45]. Notwithstanding these results, peak radial strain has shortcomings as an indicator for viability, as the sensitivity and specificity were only 33 and 72 % respectively, compared with MRI with delayed enhancement [46]. Peak strain values might be underestimated in a dyssynchronous ventricle, as both pre- and rebound stretch would decrease the absolute peak value.

**Fig. 4:**
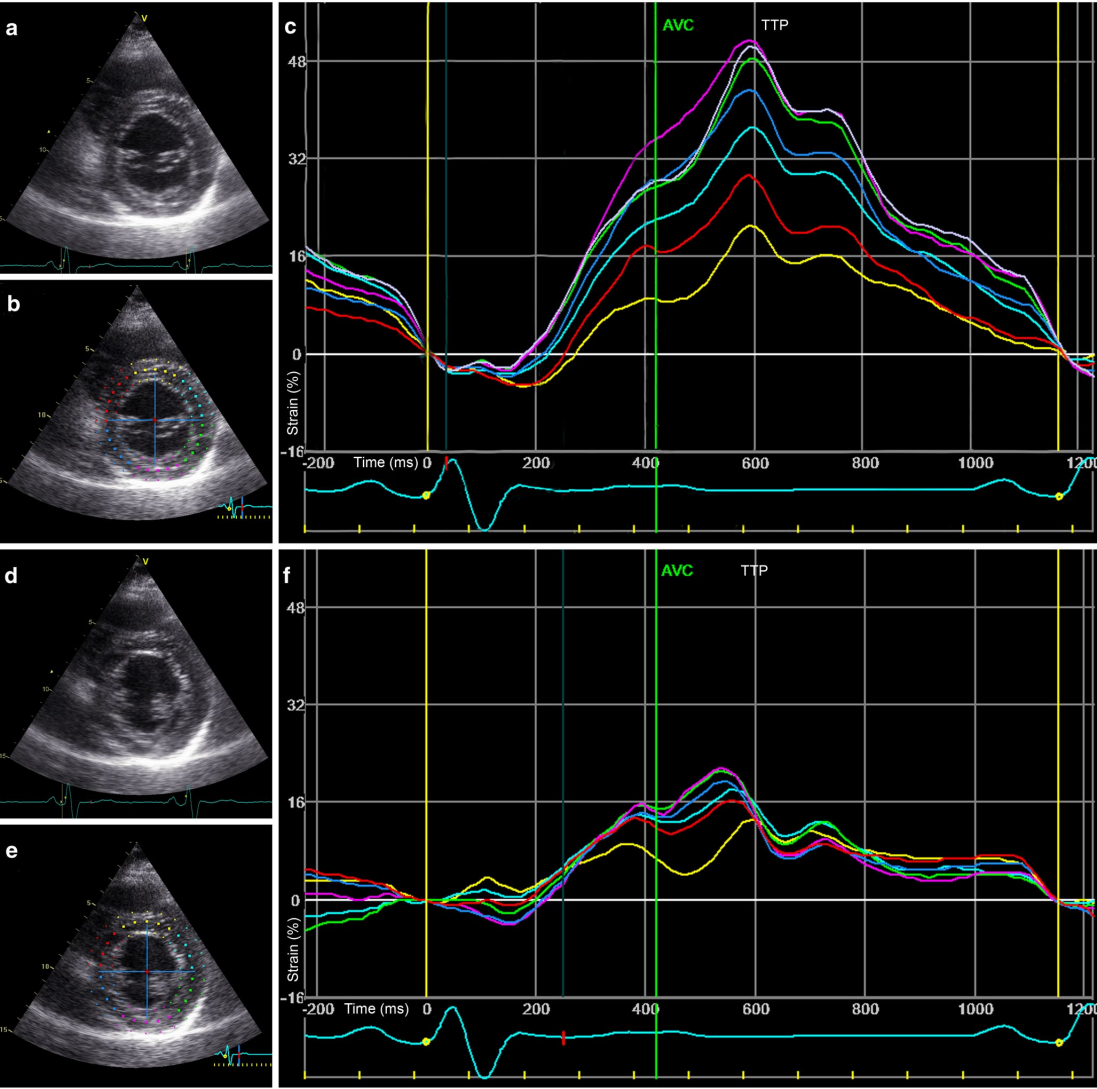
Example of echocardiographic data obtained from a responder to CRT. Apical four-chamber view, colour Doppler, septal strain and pulsed-wave Doppler acquisition of a responder to CRT, before, and 6 and 12 months after implantation. Note the continuous decrease in LV volume, decrease in mitral regurgitation, improvement in septal strain and decrease in IVMD over time. These data suggest a continuous process of reverse remodelling. Septal strain: *yellow*, *light blue* and *green* lines represent basal, mid and apical inferoseptal segmental strain, respectively. The three curves represent the segments illustrated in baseline echocardiogram in the *upper left* panel. The *white dashed* curve represents the average septal strain. SRSsept is marked *red*, as all rebound stretch after initial shortening, during systole. IVMD is represented by pulsed-wave Doppler signals of the *left* and *right* ventricular outflow tract. *EDV* end-diastolic volume, *ESV* end-systolic volume, *EF* ejection fraction, *ΔESV* change in ESV compared with baseline, *SRS* systolic rebound stretch, *LVPEP left* ventricular pre-ejection period, *RVPEP right*-ventricular pre-ejection period, *IVMD* interventricular mechanical delay. Volumes are derived by biplane Simpson’s method

High image quality is important for reliable strain analysis, which is relatively difficult to obtain in typical CRT patients with dilated left ventricles. Segments distal from the echo probe (i.e. basal and mid-inferoseptal, inferior and inferolateral segments) are prone to noise, and therefore result in more random strain curves. Even if quality is sufficient, time-to-peak strain values can be quite comparable between segments, as can be appreciated in Fig. 4. Using longitudinal strain could be a solution, it has more pronounced regional differences and is more robust than radial strain [47]. Lastly, loading conditions need to be identical between recordings, which means that changes in heart rhythm disturb the result. 3DE could be an answer to the above-mentioned difficulties, assessing the entire left ventricle in a single recording, although current techniques require multiple consecutive beats [48].

## Optimisation of device configuration

Echocardiography can be used to optimise atrioventricular and/or interventricular delays (AV/VV delay). AV-delay optimisation influences ventricular filling and may cause fusion with intrinsic conduction, thereby also influencing intraventricular and interventricular interaction. VV-delay optimisation also influences intraventricular and interventricular dynamics, leading to more homogenous LV contraction. Optimisation methods used in previous trials are: iterative or Ritter method of mitral valve inflow characteristics, velocity time integral of Doppler echocardiography of LVOT, and dyssynchrony indices using visual assessment, speckle tracking echocardiography or tissue Doppler imaging. Optimisation influences acute haemodynamic and mechanical interaction [49]. Van Deursen et al. showed the interaction between electrocardiography, strain analysis by speckle tracking echocardiography, IVMD, velocity time integral of the LVOT, and blood pressure based on finger plethysmography, while adjusting either the AV or VV delay in CRT patients [49].

Optimisation has comparable results on long-term outcome to standard or fixed delays [50]. The SMART-AV study, for example, showed no benefit of echocardiographic optimisation with an iterative method compared with a fixed AV delay of 120 ms [2]. Although Mullens et al. showed that 47 % of clinical non-responders (no significant New York Heart Association (NYHA) class or 6-minute walking test improvement) had a suboptimal AV delay, no trials so far have been published on the effects of optimisation on change in volumetric response [51]. Except for the unpublished RESPONSE-HF study, which showed no benefit of VV-delay optimisation in non-responders in a preliminary report [50]. All echocardiographic optimisation methods optimise relatively small changes (10–20 ms difference in AV and/or VV delay) with a parameter prone to noise. Patient repositioning, breathing, echocardiographic probe displacement, and other physiological disturbances all influence results. Even if one were to overcome the first three, reliable and reproducible measurement requires numerous iterations [52, 53]. This implies even more time-consuming protocols, which are unlikely to be used by clinicians. Moreover, blinding observers for settings using the iterative method leads to an even larger spread in optimums compared with unblinded optimisation, suggesting a significant amount of observer bias [47]. The optimal AV delay can also differ during different physiological conditions (i.e. rest and exercise) and may change due to remodelling [54, 55]. These limitations currently make echocardiography unsuitable for optimisation.

## Defining response with echocardiography

For response prediction, an echocardiogram is performed at least 6 months after CRT implantation, to compare volumetric status pre- and post-implantation (after a period of preferred reverse remodelling). Reverse remodelling is characterised by a decrease in LV volume. A ≥ 15 % decrease in LV end-systolic volume is commonly used to define response to CRT, or if lower, non-response. Although clinical response to CRT is multifactorial and is observed in patients without remodelling, volumetric change (i.e. decrease in end-systolic volume) predicts clinical response and prognosis of CRT patients. A larger decrease in end-systolic volume means fewer hospitalisations for heart failure and a lower mortality rate. When divided into subgroups (negative, non-responder, normal, and super-responder), there is an upscaling effect. Super-responders had almost no events during 5 years of follow-up, while non and negative responders have progressive heart failure and subsequent events [56]. Moreover, end-systolic volume decrease is preferred over clinical parameters (NYHA class, 6-minute walking distance, and quality of life score [57]. Quality of life and reverse remodelling do not always overlap, as patients can show improvement in one without the other [58]. Clinical parameters should not be disregarded, as an increase in quality of life or NYHA class can be as important to a patient as survival. Health status responders, defined by the Kansas City Cardiomyopathy Questionnaire (KCCQ) score, have a 76 % lower risk of subsequent events [58]. A reason for the missing link could be the time of volumetric assessment [59]. Studies have shown that reverse remodelling is a continuous process, with patients still showing improvement after a year of CRT (Fig. 3; [28]). Patients who are below the threshold of response at 6 months could become responders afterwards. Even patients with a proven response can have a reversal in effect, as volumetric assessment 14 months after initial response can show an increase in end-systolic volume to pre-CRT levels [60]. Assessment after 14 months of CRT proved to be a better predictor of major adverse cardiac events. These results necessitate a continuous re-evaluation of volumetric status.

## Future directions

Echocardiography could play a larger role in CRT, especially if 3D acquisition were to become more feasible for clinical practice. Increased spatial and temporal resolution could make 3DE applicable for fast acquisition of volumetric status and myocardial strain [14, 39]. A single acquisition could therefore incorporate information on function, volume, viability, dyssynchrony, and options for LV lead positioning.

Although validation in a large multicentre trial is required, dyssynchrony parameters based on patterns (i.e. SRSsept and strain patterns) instead of timing are promising for response prediction. These indices define a treatable substrate of dyssynchrony and have shown their predictive value in several studies [31, 35].

Patients with LVEF > 35 % (especially in patients with LBBB) are an interesting group for further research. These individuals might benefit from CRT. Whether their LV volumes should be determined by echocardiography or MRI and whether guidelines should distinguish between the technique used, also requires further study. Lastly, measurement variability should be addressed for reliable assessment. Measuring and averaging multiple beats should be common practice, although not all software tools incorporate average values for volume and ejection fraction (i.e. Xcelera, Philips).

## Conclusion

Echocardiography is a practical clinical tool and used for assessment of volumetric status, function and outcome in CRT patients (central illustration). Clinicians should know its shortcomings when implementing results in clinical decision making. Specific cut-off values determined by a less accurate technique require a lenient approach. So far, response prediction and patient selection by mechanical dyssynchrony parameters have been disappointing. However a new set of parameters (i.e. deformation imaging) has shown promising results in single-centre studies and requires a multicentre approach to prove its benefit. While implementation of AV or VV delay optimisation is difficult with current techniques, deformation parameters may guide LV lead placement, increasing response rates and improving prognosis. We therefore conclude that echocardiography is a friend of CRT, with known limitations. Their relationship could become even stronger, with promising applications of 3DE and deformation imaging to patient selection and optimisation of lead placement.

### Funding

None.

### Conflict of interests

None declared.
