# Erratum to: Echocardiography and cardiac resynchronization therapy, friends or foes?

**DOI:** 10.1007/s12471-016-0807-9

**Published:** 2016-02-17

**Authors:** W. M. van Everdingen, J. C. Schipper, J. van ’t Sant, K. Ramdat Misier, M. Meine, M. J. Cramer

**Affiliations:** Department of Cardiology, University Medical Centre Utrecht, Heidelberglaan 100, 3508 GA PO Box 85500, Utrecht, The Netherlands

**Erratum to:** Neth Heart J (2016) 24:25–38

DOI 10.1007/s12471-015-0769-3

Figures 3 and 4 and the captions of figures 2, 3, and 4 in the original article were incorrect and should have appeared as presented in this erratum. The editors apologise for this oversight and any confusion this may have caused.

**Fig. 2:**
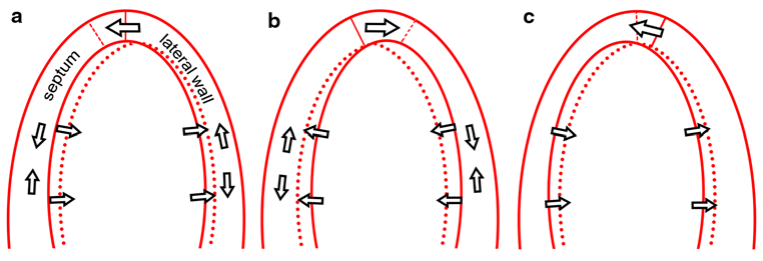
Schematic representation of apical rocking and septal flash. Schematic representation of the *left* ventricle in echocardiographic AP4CH view, showing both septal flash and apical rocking due to LBBB induced mechanical dyssynchrony. **a** early septal contraction stretches the lateral wall and rocks the apex to the *left*, while the septum thickens and moves inwards. **b** late lateral wall contraction stretches the septum and rocks the apex to the *right*. **c** relaxation of the lateral wall with continuing septal contraction, while the apex moves to its original position.

**Fig. 3:**
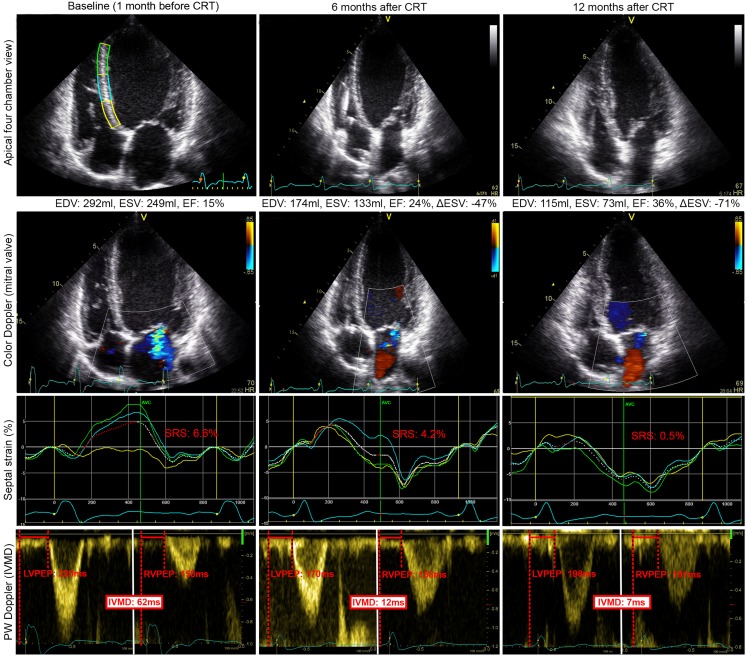
Example of echocardiographic data obtained from a responder to CRT. Apical four chamber view, colour Doppler, septal strain and pulsed-wave Doppler acquisition of a responder to CRT, before, and 6 and 12 months after implantation. Note the continuous decrease in LV volume, decrease in mitral regurgitation, improvement in septal strain and decrease in IVMD over time. These data suggest a continuous process of reverse remodelling. Septal strain: *yellow, light blue* and *green lines* represent basal, mid and apical inferoseptal segmental strain, respectively. The three *curves* represent the segments illustrated in baseline echocardiogram in the *upper left* panel. The *white dashed curve* represents the average septal strain. SRSsept is marked *red*, as all rebound stretch after initial shortening, during systole. IVMD is represented by PW-Doppler signals of the *left* and *right* ventricular outflow tract. *EDV* end-diastolic volume, *ESV* end-systolic volume, *EF* ejection fraction, *∆ESV* change in ESV compared to baseline, *SRS* systolic rebound stretch, *LVPEP* left ventricular pre-ejection period, *RVPEP* right-ventricular pre-ejection period, *IVMD* inter-ventricular mechanical delay. Volumes are derived by biplane Simpson method.

**Fig. 4:**
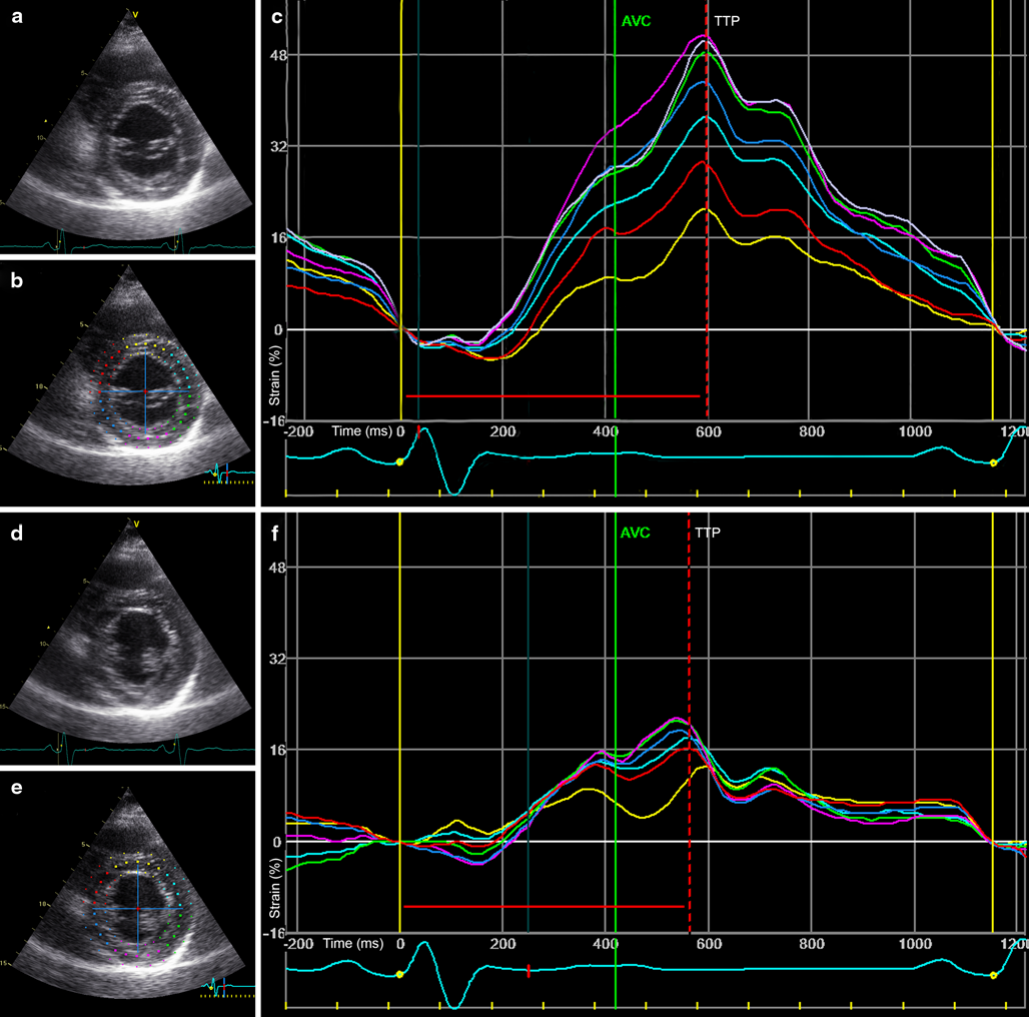
Radial strain analysis of parasternal short axis images. Parasternal short axis (PSAX) views and radial strain analysis of a patient with LBBB, imaged prior to CRT implantation. LV lead placement resulted in a mid-posterolateral position (*green curve* in Fig. 4f). ESV decrease after 6 months of therapy was 49 % (88–43 ml). **a** & **d** B-mode images with excellent echocardiographic quality of PSAX mitral valve level and papillary muscle level. **b** & **e** region of interest (ROI) placement for radial strain analysis of both PSAX views. **c** & **f** strain curves of corresponding ROIs; note the similarity in time-to-peak strain. There is no single area with latest activation.

